# Novel 1,3,4-oxadiazole Targets STAT3 Signaling to Induce Antitumor Effect in Lung Cancer

**DOI:** 10.3390/biomedicines8090368

**Published:** 2020-09-21

**Authors:** Vikas H. Malojirao, Swamy S. Girimanchanaika, Muthu K. Shanmugam, Ankith Sherapura, Dukanya, Prashant K. Metri, Vellingiri Vigneshwaran, Arunachalam Chinnathambi, Sulaiman Ali Alharbi, Shobith Rangappa, Chakrabhavi Dhananjaya Mohan, Basappa, Bettadathunga T. Prabhakar, Kanchugarakoppal S. Rangappa

**Affiliations:** 1 Molecular Biomedicine Laboratory, Postgraduate Department of Studies and Research in Biotechnology, Sahyadri Science College, Kuvempu University, Shivamogga, Karnataka 577203, India; vikasvicky582@gmail.com (V.H.M.); ankithsbiotech@gmail.com (A.S.); vigsbio@gmail.com (V.V.); 2 Laboratory of Chemical Biology, Department of Studies in Organic Chemistry, University of Mysore, Manasagangotri, Mysore, Karnataka 570006, India; swamynayak010@gmail.com (S.S.G.); dukanya4@gmail.com (D.); prashant.metri@gmail.com (P.K.M.); 3 Department of Pharmacology, Yong Loo Lin School of Medicine, National University of Singapore, Singapore 117597, Singapore; phcsmk@nus.edu.sg; 4 Department of Pharmacology, University of Illinois at Chicago, Chicago, IL 60612, USA; 5 Department of Botany and Microbiology, College of Science, King Saud University, Riyadh 11451, Saudi Arabia; carunachalam@ksu.edu.sa (A.C.); sharbi@ksu.edu.sa (S.A.A.); 6 Adichunchanagiri Institute for Molecular Medicine, AIMS Campus, B. G. Nagar, Nagamangala Taluk, Mandya District 571448, India; shobithrangappa@gmail.com; 7 Department of Studies in Molecular Biology, University of Mysore, Manasagangotri, Mysore, Karnataka 570006, India; cd.mohan@yahoo.com; 8 Institution of Excellence, Vijnana Bhavan, University of Mysore, Mysore 570006, India

**Keywords:** lung cancer, antitumor, STAT3, apoptosis

## Abstract

Lung cancer is the leading type of malignancy in terms of occurrence and mortality in the global context. STAT3 is an oncogenic transcription factor that is persistently activated in many types of human malignancies, including lung cancer. In the present report, new oxadiazole conjugated indazoles were synthesized and examined for their anticancer potential in a panel of cancer cell lines. Among the new compounds, 2-(3-(6-chloro-5-methylpyridin-3-yl)phenyl)-5-(1-methyl-1H-indazol-3-yl)-1,3,4-oxadiazole (CHK9) showed consistently good cytotoxicity towards lung cancer cells with IC_50_ values ranging between 4.8–5.1 µM. The proapoptotic effect of CHK9 was further demonstrated by Annexin-FITC staining and TUNEL assay. In addition, the effect of CHK9 on the activation of STAT3 in lung cancer cells was examined. CHK9 reduced the phosphorylation of STAT3^Y705^ in a dose-dependent manner. CHK9 had no effect on the activation and expression of JAK2 and STAT5. It also reduced the STAT3-dependent luciferase reporter gene expression. CHK9 increased the expression of proapoptotic (p53 and Bax) proteins and decreased the expression of the antiapoptotic (Bcl-2, Bcl-xL, BID, and ICAM-1) proteins. CHK9 displayed a significant reduction in the number of tumor nodules in the in vivo lung cancer model with suppression of STAT3 activation in tumor tissues. CHK9 did not show substantial toxicity in the normal murine model. Overall, CHK9 inhibits the growth of lung cancer cells and tumors by interfering with the STAT3 signaling pathway.

## 1. Introduction

Lung cancer is the leading cause of cancer-related mortality worldwide, accounting for approximately 25% of all cancer deaths [1,2]. Non-small cell lung carcinoma and small cell lung carcinoma are the major types of lung cancer with the occurrence rate of 85% and 15%, respectively [3,4]. Smoking, exposure to asbestos, and inhalation of gaseous mutagens are the important risk factors associated with the initiation of lung cancer [5,6]. Lung cancer can be treated using surgical approaches, including resection of the affected part of the liver and non-surgical strategies such as biologic therapy, radiotherapy, and chemotherapy [7,8]. The five-year survival rate of lung cancer patients is as low as 18.6%, and it increases to 56.3% if the cancer is treated when it is localized and before it attains the metastatic phenotype [9]. Therefore, early detection and treatment of lung cancer may enhance the overall survival rate of lung cancer patients. 

Signal transducer and activator of transcription (STAT) proteins are transcription factors that reside in the cytoplasm of mammalian cells. STAT family encompasses seven structurally related proteins, including STAT1, STAT2, STAT3, STAT4, STAT5a, STAT5b, and STAT6 [10,11,12,13]. Among the STAT forms, STAT3 and STAT5 have been extensively studied in the cancer context [14,15,16]. STAT3 is an oncogenic protein that is overactivated in many types of malignancies, including cancers of blood cells, lung, liver, breast, kidney, ovary, and colon [17,18,19,20]. In healthy cells, IL-6 transiently triggers the phosphorylation of gp130, Janus kinases (JAKs), and STAT3. The phosphorylated STAT3^Y705^ monomer undergoes dimerization with the other phosphorylated monomeric STAT3 to translocate into the nucleus [21,22,23]. The STAT3 dimer interacts with the promoter region of target genes and regulates their expression [24,25]. The signals generated by IL-6 stimulation are desensitized by the negative modulators [26,27,28]. In cancers, STAT3 is persistently activated due to the disruption of a negative regulatory system, which results in the overexpression of oncogenic proteins [28,29]. In addition, EGFR also activates STAT3 in a JAK-independent mechanism [13]. Overactivation of EGFR is commonly observed in lung cancers, which lead to the deregulated activation of STAT3 and thereby contribute to the tumor progression [30,31]. Therefore, targeting STAT3 signaling may provide significant clinical benefit in the treatment of STAT3-positive lung cancer patients.

Oxadiazole is a five-membered heterocyclic ring that possesses two nitrogen atoms and an oxygen atom in its structure [32]. Several structural derivatives of oxadiazole have been studied extensively for their cytotoxic activity and shown to possess potent anticancer activity in a wide range of cancer cells [33,34]. Recently, ODZ10117, a 1,2,4-oxadiazole derivative, was identified as the direct inhibitor of STAT3 using structure-based computational database screening and cell-based high-throughput screening [35]. ODZ10117 was reported to induce potent anticancer activity in breast cancer preclinical models by targeting the SH2 domain of the STAT3 [35]. MD77, a new 1,2,5-oxadiazole, was presented as an inhibitor STAT3 by directly interacting with the SH2 domain of STAT3 [36]. The new Pt(II) complex bearing 1,2,5-oxadiazole selectively interacted with STAT3 over STAT1 and displayed good antitumor activity in in vivo cancer model [37]. In addition, indazole derivatives were identified as the inhibitors of JAKs, which is an immediate upstream kinase of STAT3 signaling [38,39]. Therefore, we hypothesized that the conjugation of oxadiazole with indazole might yield new derivatives that are endowed with STAT3 signaling inhibitory activity. Thirteen new oxadiazole–indazole hybrids were synthesized and we evaluated their anticancer and STAT3-inhibitory potential in lung cancer cells in vitro and in vivo. 

## 2. Results

### 2.1. Chemistry

**Scheme 1:**
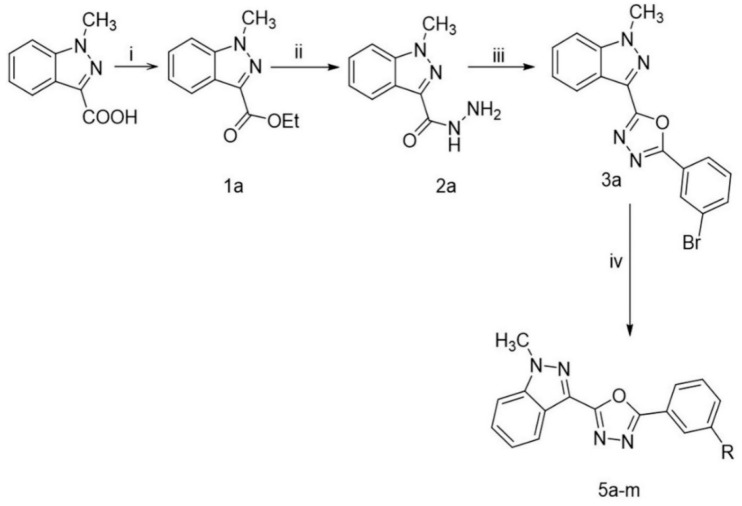
Synthetic route for the synthesis of 2-([substituted]-3-yl)-5-(1-methyl-1H-indazol-3-yl)-1,3,4-oxadiazole: Reaction Conditions: (i) Aromatic acid (1 eq), Cat.H_2_SO_4_ in 20 mL EtOH,10 h reflux (ii) ester (1 eq), hydrazine hydrate (1 eq) in 10 mL EtOH, 6 h reflux (iii) hydrazide (1 eq), acid (1 eq) in 1 mL POCl_3_, 10 h, reflux (iv) oxadiazole (1 eq), boronic acids **4a–m** (1.2 eq) K_2_CO_3_(3 eq), Pd(dppf)_2_Cl_2_ (0.1 eq),1,4-dioxane:H_2_O:EtOH (5:1:1), N_2_, 120 °C for 45 min.

1-methyl-1H-indazole-3-carboxylic acid was converted into its corresponding ester (**1a**) followed by refluxing with hydrazine hydrate in ethanol, which resulted in the formation of acid hydrazide (**2a**). Furthermore, 1,3,4-oxadiazole was synthesized by refluxing an equimolar mixture of acid hydrazide (**2a**) and 3-bromobenzoic acid in the presence of phosphorous oxychloride as a cyclizing agent (5 mL) for 10 h, which gave indazole-conjugated oxadiazole (**3a**). Then, these compounds were treated with different boronic acids to yield biphenyls (**5a–m**), as described in Scheme 1. The structures of all the target compounds (Table 1) were characterized by LCMS, ^1^H NMR, and ^13^C NMR spectrometry. 

### 2.2. Biology 

#### 2.2.1. CHK9 Induces Cytotoxicity in Lung Cancer Cells 

**Figure 1:**
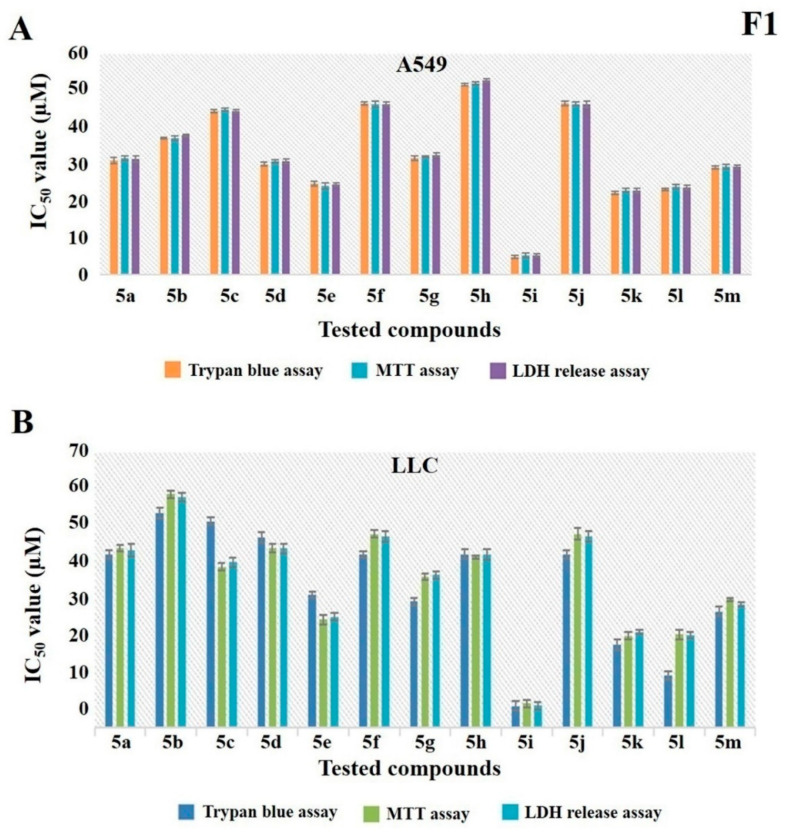
5i (CHK9) is a lead compound among other structural analogs. (**A**,**B**) A549 and Lewis lung carcinoma (LLC) cells were treated with different concentrations of all the new compounds and examined for their cytotoxic effect using trypan blue, 3-(4,5-dimethylthiazole-2yl)-2,5-diphenyltetrazolium bromide (MTT), and lactate dehydrogenase (LDH) release assay. IC_50_ values were calculated for each compound in all the assay systems and the graph is plotted.

We initially examined the cytotoxic effect of new compounds towards a panel of cancer cell lines of different tissue origin including human lung cancer (A549), breast cancer (MCF-7), skin cancer (A375), liver (HepG2, Huh-7), kidney cancer (ACHN, A498) and mouse skin cancer (B16-F10) and Lewis lung carcinoma (LLC) cell lines using 3-(4,5-dimethylthiazole-2yl)-2,5-diphenyltetrazolium bromide (MTT) reduction, lactate dehydrogenase (LDH) leakage, and trypan blue dye exclusion assay systems. Among the new oxadiazole derivatives, CHK9 (compound 3i) displayed consistent cytotoxicity towards all the tested cell lines in all the three assay systems. The IC_50_ values of CHK9 are A549 ≥ 5 μM, LLC ≥ 5 μM, MCF-7 ≥ 7.27 μM, HepG2 ≥ 11.3 μM, B16F10 ≥ 14μM, A375 ≥ 16.43 μM, ACHN ≥ 18.43 μM, A498 ≥ 21 μM, and Huh7 ≥ 27.43 μM. Since the CHK9 displayed good cytotoxicity, particularly towards lung cancer cells (Figure 1A,B), we performed further experiments using A549 cells.

#### 2.2.2. CHK9 Triggers Apoptosis in Lung Cancer Cells

**Figure 2:**
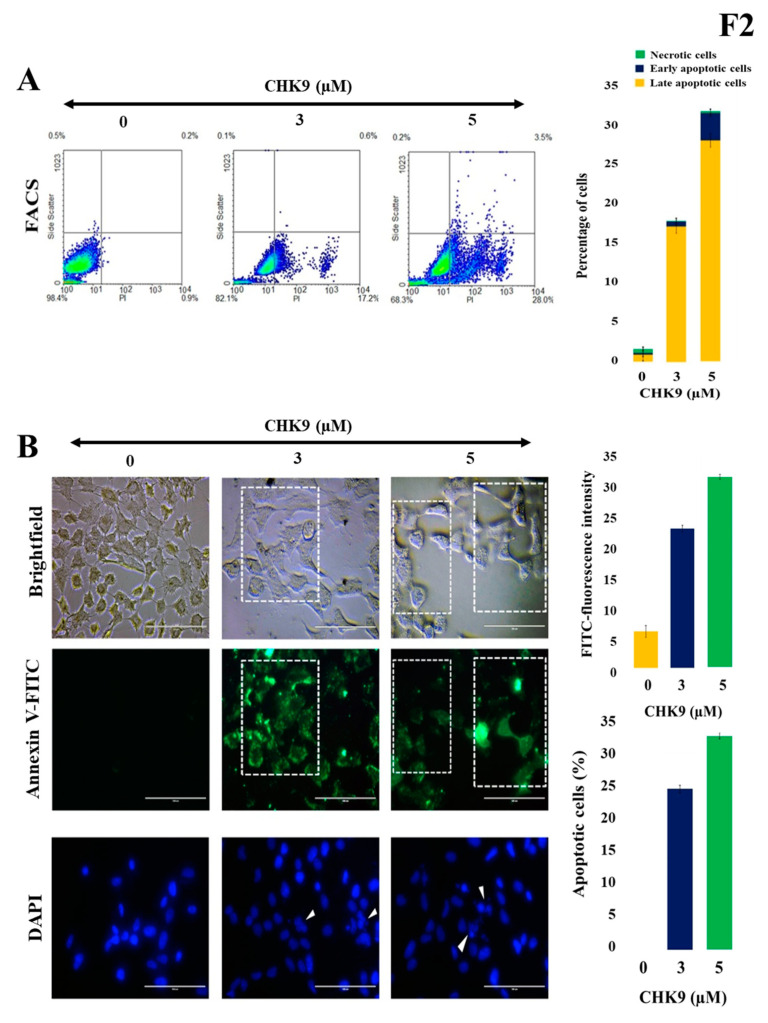
CHK9 induces apoptosis in lung cancer cells. The A549 cells were treated with CHK9 at 0, 3 and 5 µM for 48 h and used for further experiments. (**A**) Densitometric plot with a graphical representation of apoptotic cells/necrotic cells. (**B**) Annexin V immune staining images of bright field and fluorescent images and condensed nuclear changes in DAPI stained images (Magnification: 20×). The cells with shrunken or fragmented nuclei with condensed chromatin were indicated using white arrowheads.

Next, we examined whether the cytotoxic potential of CHK9 is due to the induction of apoptosis in lung cancer cells. A549 cells were treated with different concentrations of CHK9 (0, 3, and 5 µM) and analyzed the percentage of apoptotic cells using flow cytometry. The results show that CHK9 treatment increased the percentage of apoptotic cells to 17.8 and 31.7% at 3 and 5 µm, respectively. The densitometric plot and distribution of the cells are provided in Figure 2A. 

**Figure 3:**
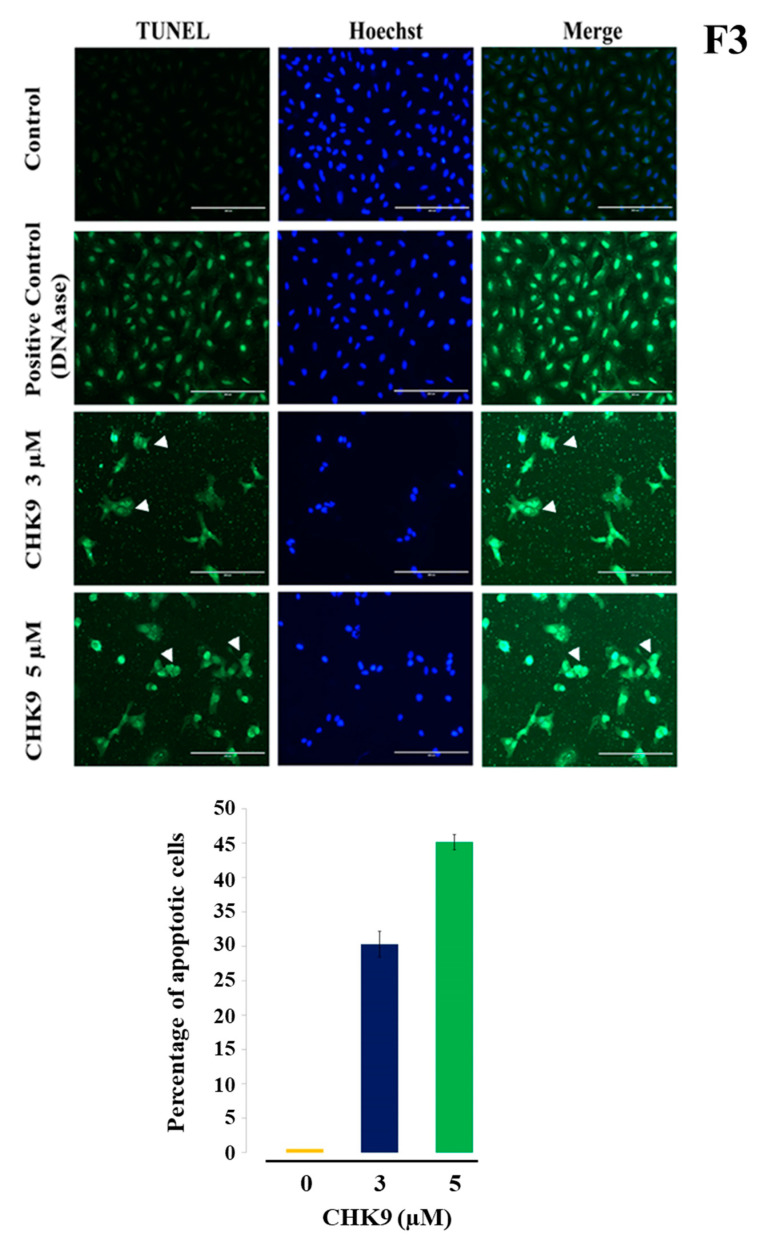
CHK9 increases TUNEL-positive cells. The A549 cells were treated with CHK9 at 0, 3 and 5 µM for 48 h and used for TUNEL assay. Hoechst staining is used for staining the nucleus. DMSO/DNase/CHK9-treated cells were photographed, quantified, and expressed in the form of a bar graph (Magnification: 20×). TUNEL-positive cells were indicated using white arrowheads.

In the next experiment, we treated A549 cells with different concentrations of CHK9 (0, 3, and 5 µM) and stained with FITC-conjugated annexin V and 4′,6-diamidino-2-phenylindole (DAPI) to visualize the apoptotic cells. We observed the increase in the green fluorescence in a concentration-dependent manner, and condensation of the nucleus was experimentally validated by DAPI staining on treatment with CHK9, indicating the increase in the apoptotic cells (Figure 2B). We next performed the TUNEL assay to reconfirm the apoptogenic potential of CHK9. The cells were treated with different concentrations of CHK9 (0, 3, and 5 µM) and analyzed for TUNEL-positive cells. The percentage of the TUNEL-positive cells was 31.3 and 46.2% at 3 and 5 µM, respectively (Figure 3). DNase was used as a positive control and counterstained with Hoechst for specific nuclear localization.

#### 2.2.3. CHK9 Specifically Inhibits STAT3 Activation without Modulating the Activity of JAK2 in Lung Cancer Cells

**Figure 4:**
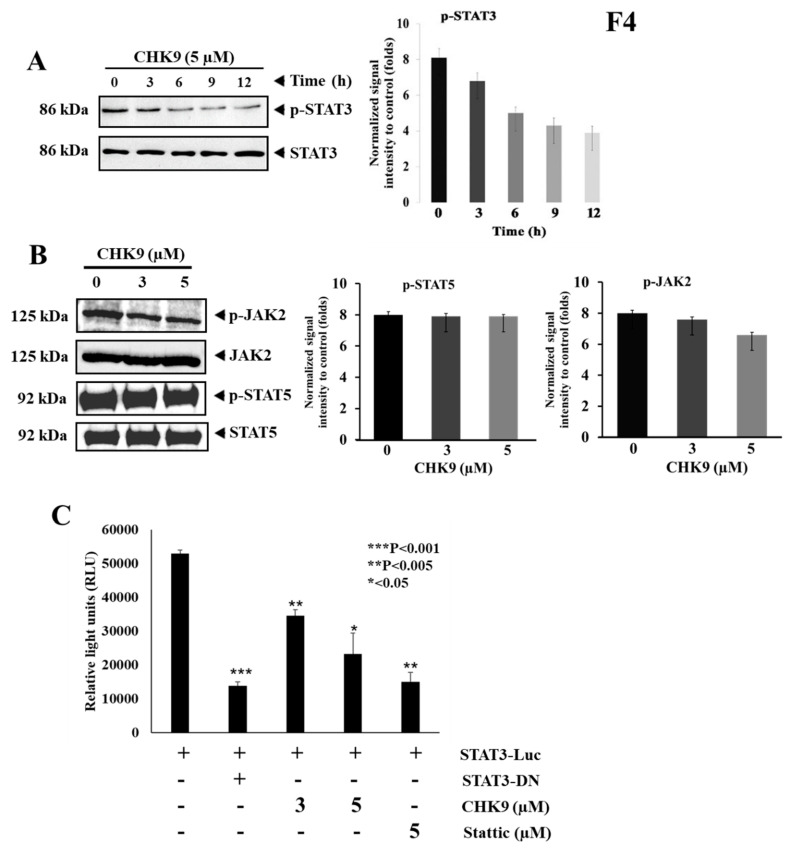
CHK9 suppresses the phosphorylation of STAT3^Y705^ without affecting JAK2. (**A**) A549 cells were treated with CHK9 (5 µM) for indicated time-points and the whole-cell lysate was prepared to use it for Western blotting to analyze the expression of phospho-STAT3, STAT3 and its graphical representation of the relative normalized intensity with respective loading controls. (**B**) A549 cells were treated with the indicated concentrations of CHK9 (3 and 5 µM) for 24 h and the whole-cell lysate was prepared to use it for Western blotting to analyze the expression of phospho-JAK2, JAK2, phospho-STAT5, and STAT5. (**C**) A549 cells were transfected with STAT3-responsive elements conjugated reporter gene and these cells were incubated with indicated concentrations of CHK9 for 24 h and examined for luciferase activity. All luciferase experiments were done in triplicate and repeated twice.

We further investigated the effect of CHK9 (5 µM) for various time points (0, 3, 6, 9, and 12 h) on the activation of STAT3 in A549 cells. We observed the significant decrease in the phosphorylation of STAT3 in a time-dependent manner without significant alteration in the levels of STAT3 (Figure 4A), suggesting that CHK9 induces cytotoxicity by abrogating the STAT3 signaling pathway in lung cancer cells. We also examined the effect of CHK9 on the activation of JAK2 at indicated concentrations and did not find a significant change in phosphorylation and expression of JAK2 (Figure 4B). We next analyzed the effect of CHK9 on the activation of STAT5 and found no change in phosphorylation of STAT5, indicating the specificity of CHK9 towards STAT3 (Figure 4B). 

#### 2.2.4. CHK9 Mitigates STAT3-Dependent Gene Expression in Lung Cancer Cells

Next, we transfected A549 cells with STAT3 target elements conjugated with luciferase gene and analyzed the expression of luciferase on CHK9 treatment. CHK9 downregulated the luciferase reporter gene expression in a concentration-dependent manner (Figure 4C), indicating that the reduction in reporter gene expression could be due to the abrogation of STAT3 phosphorylation. 

#### 2.2.5. CHK9 Decreases the Expression of STAT3-Targeted Genes and p53 in Lung Cancer Cells

**Figure 5:**
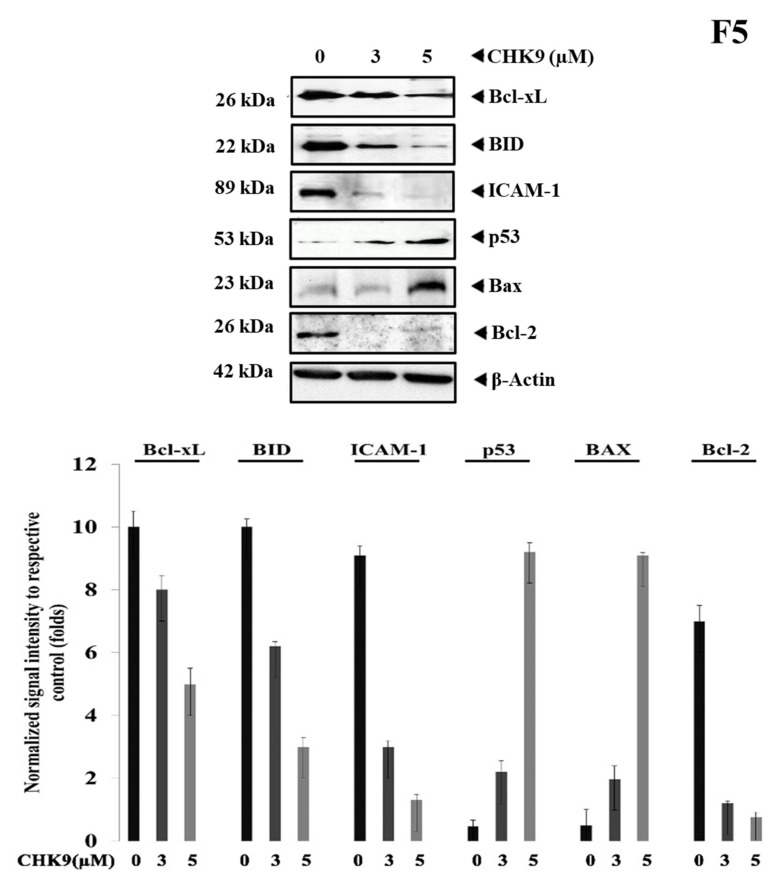
CHK9 reduced the expression of STAT3 target genes. A549 cells were treated with indicated concentrations of CHK9 for 48 h and the whole-cell lysate was prepared to use it for Western blotting to analyze the expression of Bcl-xL, BID, ICAM-1, p53, Bax, and Bcl-2. The graphical representation of the relative normalized intensity of with respective loading controls.

We investigated the effect of CHK9 on the expression of STAT3 targeted proteins (Bcl-2, Bcl-xL, ICAM-1) and other apoptosis-related proteins (Bax, BID, and p53) in lung cancer cells. CHK9 increased the expression of proapoptotic (Bax) protein and decreased the expression of STAT3-target proteins and tumor suppressor (p53) protein (Figure 5), indicating the apoptosis modulatory effect of CHK9 in lung cancer cells. 

#### 2.2.6. CHK9 Regress In Vivo Tumor Growth by Inhibiting STAT3 Signaling Cascade

**Figure 6:**
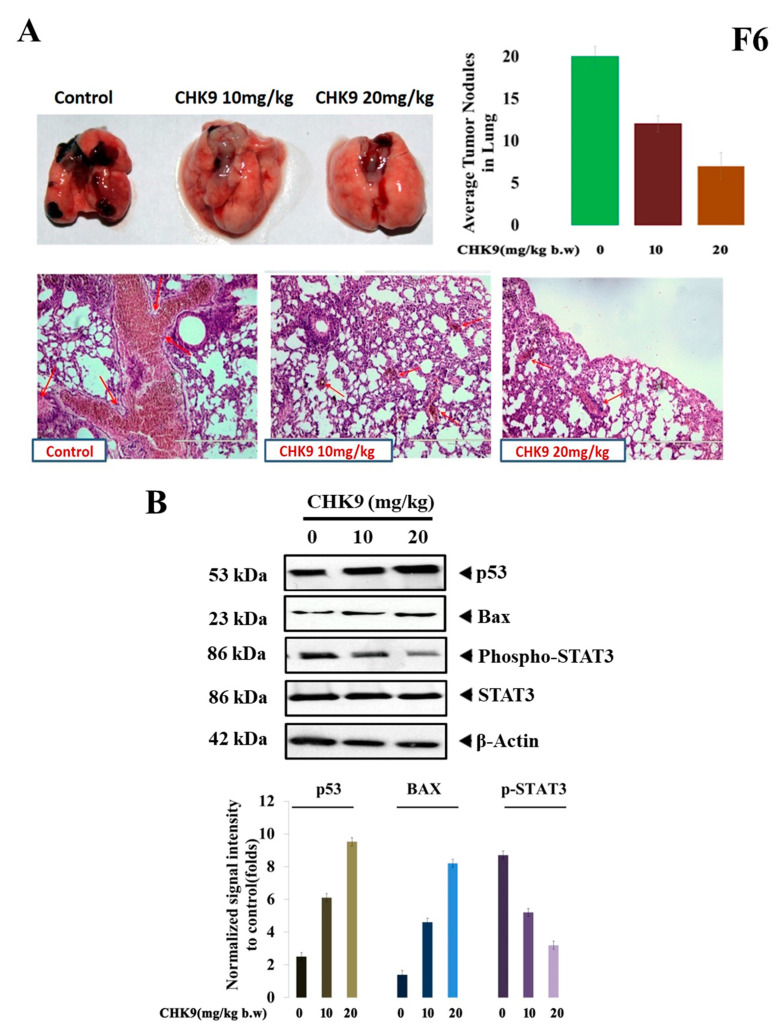
CHK9 inhibits colonization of LLC cells in animal models by inducing apoptosis. LLC cells (1 × 10^6^) were tail vein injected into the BALB/c mice. Three groups of mice received 0, 10 and 20 mg/kg of CHK9 alternative days for 30 days and after 30 days of induction, lung exhibited tumor colonization (upper panel) H&E staining of the lung tissue exhibited tumor nodules (lower panel). (**A**) Graph representing the average number of tumor nodules of each group. (**B**) Western blotting studies demonstrate the expression of p53, Bax, phospho-STAT3, STAT3, and β-actin in CHK9 treated mice in a concentration-dependent manner and graphical representation of the relative normalized intensity of with respective loading controls.

The in vivo antitumor activity of CHK9 was evaluated in an experimental lung cancer model. The CHK9 significantly decreased the lung tumor prevalence as compared to control in a dose-dependent manner (10 and 20 mg/kg). The lung tissues were observed for contracted macroscopic lung tumor multiplicity (Figure 6A). Haematoxylin and eosin (H&E) staining showed significantly lesser tumor nodules in lung tissues of the CHK9 treated mice relative to the control group. The average number of tumor nodules present in control and CHK9 (10 and 20mg/kg) treated animals was 18, 11, and 6 respectively. Furthermore, the expression of phospho-STAT3, p53, and Bax was examined in the tumor tissues derived from the experimental animals. CHK9 reduced the phosphorylation of STAT3 and increased the expression of p53 and Bax (Figure 6B), indicating that the antitumor potential of CHK9 is due to the inhibition of STAT3 signaling cascade. Notably, acute toxicity experiments revealed that CHK9 significantly did not alter the levels of blood cells, creatinine, aspartate aminotransferase, and alanine aminotransferase in normal animals (Supplementary Figure S1). 

#### 2.2.7. CHK9 Interacts with the SH2 Domain of STAT3 

**Figure 7:**
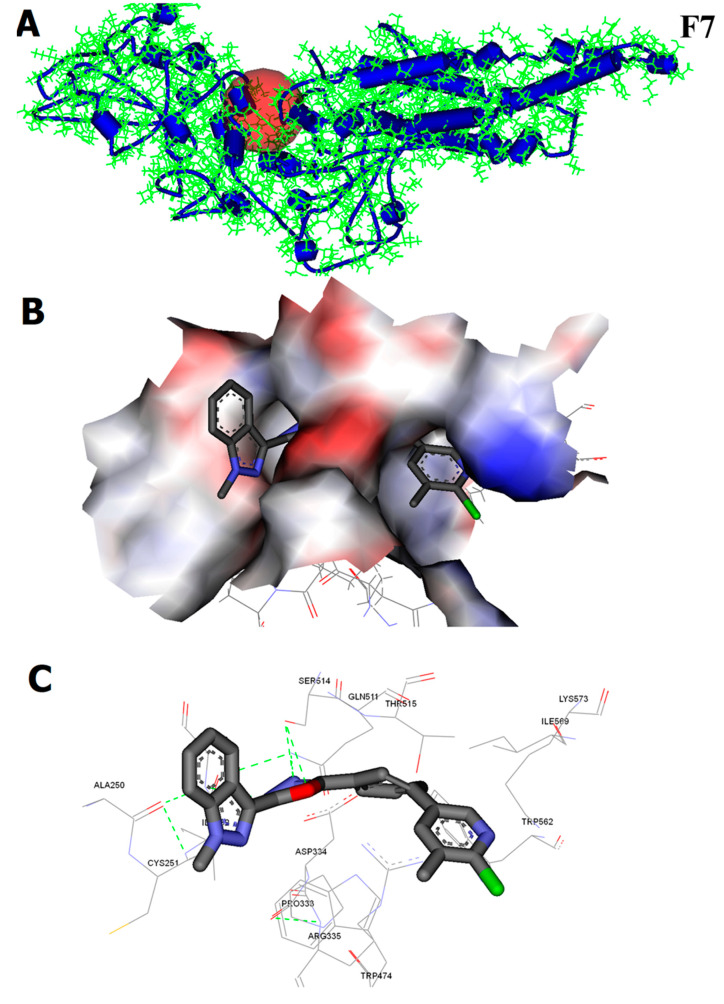
In silico interaction analysis of CHK9 binding towards the SH2 domain of STAT3. (**A**) The crystal structure of the SH2 domain of STAT3 (**B**,**C**). The surface view and interaction map of CHK9 with the SH2 domain of STAT3.

In vitro and in vivo studies showed that the CHK9 exhibits the anticancer activity by inhibiting STAT3 signaling in lung cancer cells. To understand the in silico molecular interaction of the new candidate compounds with SH2 domain of STAT3, we extracted the co-crystal structure of the DNA and STAT3β dimer and used it for molecular docking analysis (Figure 7A) as reported earlier [40]. For this purpose, the Accelrys DS version 2.5 platform was used. CDOCKER ligand-receptor interaction module was used to determine the affinity between the newly prepared structures and the SH2 domain of STAT3. The results obtained show the various degrees of docking score with the SH2 domain of STAT3. Among the tested compounds, **5a**, CHK9, **5j**, and **5m** exhibited a significant docking score with the SH2 domain. The interaction map of CHK9 with the SH2 domain of STAT3 is presented in Figure 7B,C.

## 3. Discussion

STAT3 is an oncogenic protein that transcribes the genes associated with cell proliferation, prosurvival, antiapoptosis, angiogenesis, and metastasis [41,42,43]. Persistent activation of STAT3 has been well-documented in a broad range of human malignancies, including cancers of the lung, breast, ovary, colon, gastric, head and neck, pancreatic, and kidney [44,45,46]. The relatively high expression of phosphorylated STAT3 has been observed in the tumor tissues compared to the non-diseased tumor surrounding tissues [13]. Importantly, elevated expression of activated STAT3 serves as a marker of negative prognosis in many types of cancers. Moreover, STAT3 is activated by many upstream signaling cascades, including JAKs, Src, EGFR, and other growth factors, which makes it a point of oncogenic signal convergence [47]. Therefore, targeting STAT3 using direct small molecule inhibitors serves as a good strategy to counteract the deleterious effects of STAT3 in cancer cells. Recent studies revealed that the synthetic oxadiazoles possess STAT3 inhibitory activity in various preclinical cancer models. Recently, Kim and colleagues identified 5-tert-butyl-3-(3-nitro-phenoxymethyl)-[1,2,4]oxadiazole (ODZ17690) as the inhibitor of STAT3 using a computational approach [35]. Furthermore, they used ODZ17690 as the template structure and prepared its 144 derivatives. Among the new derivatives, compound ODZ10117 was identified as the most potent inhibitor in cell-based experiments [35]. Fathi and coworkers reported the anticancer and STAT3-inhibitory activity of oxadiazole conjugated chalcones [48]. Khanam and team identified a 1,3,4-oxadiazole derivative that directly interacts with the SH2 domain of STAT3 in docking analysis and direct binding studies [49]. Collectively, these studies proposed that the conjugation of 1,3,4-oxadiazole moiety with other heterocycle yield new structures with STAT3 inhibitory activity. Herein we synthesized new derivatives of oxadiazole and indazole conjugates and tested them for possible anticancer and STAT3 inhibitory potential in lung cancer cells. Although several heterocyclic systems have been reported to inhibit STAT3 signaling, to best of our knowledge, this is the first report on the STAT3 inhibitory efficacy of oxadiazole-indazole conjugates in lung cancer model. Although CHK9 is not the best STAT3 inhibitor among the previously reported small molecules, it showed relatively good, consistent, and specific inhibition towards STAT3. 

Initial investigations on the cytotoxic potential of title compounds presented CHK9 as a lead hit, and the apoptogenic effect of CHK9 was examined by staining with propidium iodide and FITC conjugated annexin-V. Propidium iodide permeates the non-viable cells and stains their fragmented DNA, which can be detected in flow cytometry [50]. The phosphatidylserine is generally located in the inner leaflet of the biological membrane and translocates into the outer leaflet during apoptosis, which can be stained using FITC-labelled annexin V [51]. The treatment with CHK9 enhanced the propidium iodide and annexin V-stained cells, indicating the cells are undergoing apoptosis. The triggering of apoptotic signaling results in the activation of caspases and DNases that lead to the fragmentation of the genomic DNA. This event can be detected using an enzyme terminal deoxynucleotidyl transferase, which transfers fluorescently labeled deoxynucleotides to the 3’-end of cleaved DNA. CHK9 cells displayed a higher intensity of fluorescence demonstrating that CHK9 induces apoptosis. Based on the previous reports, the effect of CHK9 on STAT3 activation in lung cancer cells was investigated and CHK9 was found to decrease the phosphorylation of STAT3^Y705^ in the tested cell lines. STAT3^Y705^ phosphorylation is critical for its dimerization and translocation into the nucleus to express target genes [52,53]. Therefore, inhibition of STAT3 phosphorylation by CHK9 may result in the abrogation of STAT3 downstream signaling events. Among the STAT variants, STAT3 and STAT5 are well-studied in cancer relevance and frequently observed to undergo constitutive activation in several human cancers. Our investigation revealed that CHK9 does not affect either activation or expression of STAT5, indicating that CHK9 selectively inhibits STAT3 phosphorylation. JAK2 is a major upstream nonreceptor tyrosine kinase that activates STAT3 and we investigated the effect of CHK9 on the activation of JAK2 in lung cancer cells. Interestingly, CHK9 did not inhibit JAK2 phosphorylation, indicating that CHK9 is not interfering with the activity of upstream JAKs. In addition, we tested the effect of CHK9 on STAT3-directed transcription of the luciferase reporter gene. The transfected cells displayed high luciferase activity which was reversed by the use of dominant-negative STAT3 and the same effect was observed when transfected cells were treated with CHK9. This is a piece of direct evidence to demonstrate that CHK9 inhibits STAT3 and thereby blocks STAT3-mediated transcription. The profiling of expression of STAT3 target genes such as Bcl-2, Bcl-xL, ICAM-1, and BID revealed the decline in their expression upon CHK9 treatment. This observation was in agreement with the results of the inhibition of STAT3 phosphorylation and reporter gene expression studies. 

STAT3 and p53 have counteracting functional roles—the former is oncogenic, and the latter is tumor-suppressor protein. Reports suggested that STAT3 interacts with the promoter of p53 to suppress its expression in mouse cell lines [54]. CHK9 upregulated the expression of p53 and antiapoptotic (Bax) protein indicating the triggering of apoptotic response in lung cancer cells. In support of these results, CHK9 decreased the expression of the antiapoptotic protein (Bcl-2). In addition, in silico interaction studies showed that CHK9 interacts with the SH2 domain of STAT3 with relatively good docking scores, and these results are on par with the findings of cell-based experiments.

Moreover, CHK9 did not exhibit toxicity up to 20 mg/kg body weight in the tested animals. 1,3,4-oxadiazoles have displayed similar toxicity profiles in the previous in vitro and in vivo studies [55,56]. A limited number of studies have demonstrated the in vivo antitumor activity of oxadiazoles. Tiwari and colleagues reported that four 1,3,4-oxadiazoles decrease the tumor volume in Dalton’s lymphoma ascites-inoculated mice [57]. CHK9 displayed good antitumor activity in lung cancer model with depletion of activated STAT3 and other oncogenic proteins in tumor tissues. In conclusion, CHK9 exhibits growth-inhibitory potential by abrogating STAT3 signaling in lung cancer cells.

## 4. Materials and Methods

### 4.1. Chemistry

#### 4.1.1. Chemicals, Reagents, and Instruments

The advancement of the reaction was detected using thin-layer chromatography (TLC). Analytical TLC was performed on pre-coated Merck silica gel 60 F254 plates using ethyl acetate and hexane as eluent, and the spots were detected under UV light. ^1^H NMR and^13^C NMR spectra were recorded on Agilent NMR instrument in CDCl_3_ solvent. Chemical shifts were expressed in ppm downfield relative to the TMS. Mass spectra were recorded on an Agilent LC-MS. All solvents and reagents were commercially available reagent grade. Column chromatography separations were obtained on silica gel (60–120 mesh).

#### 4.1.2. Synthesis of ethyl 1-methyl-1H-indazole-3-carboxylate (**1a**)

A mixture of 1-methyl-1H-indazole-3-carboxylic acid (10 g, 0.057 moles), anhydrous ethanol (40 mL), and concentrated sulfuric acid (1 mL) were taken in a round bottom flask and refluxed for 6 h. Excess of ethanol was distilled off, and the contents were transferred to a separating funnel containing 50 mL of distilled water. The synthesized ester was extracted three times with 50 mL chloroform. The organic layer was washed with brine solution and dried over sodium sulfate. Chloroform was distilled off under reduced pressure to get the desired ester (**1a**), which was weighed after drying. 

#### 4.1.3. Synthesis of 1-methyl-1H-indazole-3-carbohydrazide (**2a**)

A mixture of ethyl 1-methyl-1H-indazole-3-carboxylate (8 g, 0.039 moles), hydrazine hydrate (1.9 mL, 0.039 moles) and 30 mL of ethanol was taken in a round bottom flask and refluxed for 6 h. Ethanol and excess of hydrazine hydrate were distilled off under reduced pressure, and after quenching with excess ice-cold water, the white-colored precipitate was filtered off and recrystallized in ethanol.

#### 4.1.4. Synthesis 2-(3-bromophenyl)-5-(1-methyl-1H-indazol-3-yl)-1,3,4-oxadiazole (**3a**)

A mixture of 1-methyl-1H-indazole-3-carbohydrazide (8 g, 0.042 moles), 3-bromobenzoicacid (8.4 g, 0.042 moles) and phosphorus oxychloride (5 mL, 0.13 moles) was refluxed for 8 h, cooled and poured on crushed ice. The solid separated was filtered, treated with potassium carbonate, washed with water and purified by column chromatography using ethyl acetate and hexane as eluent.

#### 4.1.5. General Procedure for the Synthesis of Compounds (**5a–m**)

A mixture of ethyl 2-(3-bromophenyl)-5-(1-methyl-1H-indazol-3-yl)-1,3,4-oxadiazole(**3a**)(1 eq), and different boronic acids (**4a**–**m**) (1.2 eq), potassium carbonate(3 eq), [1,1’-bis(diphenylphosphino)ferrocene]dichloropalladium(II) (0.1 eq) were taken in seal-tube. 1,4-dioxane, water, and ethanol in 5:1:1 ratio were added under a nitrogen atmosphere, and the mixture was heated at 120 °C for 45 min. The completion of the reaction was monitored by TLC, and further, it was purified by column chromatography using ethyl acetate and hexane as eluent.

#### 4.1.6. 2-(1-methyl-1H-indazol-3-yl)-5-(3-(pyridin-3-yl)phenyl)-1,3,4-oxadiazoles (**5a**)

Off-white solid; mp139–142 °C; 82% yield; ^1^H NMR (CDCl_3_, 400 MHz) δ: 8.93(s,1H,Ar-H), 8.64(d,1H,*J* = 4 Hz Ar-H), 8.43(s,2H,Ar-H), 8.27(d,1H,*J* = 8 Hz,Ar-H), 7.95(d,1H,*J* = 8 Hz,Ar-H), 7.77(d,1H,*J* = 8 Hz,Ar-H), 7.66(t,1H,*J* = 8 Hz,Ar-H), 7.49–7.55(m,2H,Ar-H), 7.37–7.44(m,2H,Ar-H), 3.74(s,3H,N-CH_3_), MS: 353.38 m/z =354.03 [M + 1]^+^. 

#### 4.1.7. 2-(3’-methoxy-[1,1’-biphenyl]-3-yl)-5-(1-methyl-1H-indazol-3-yl)-1,3,4-oxadiazole (**5b**)

White solid; mp140–145 °C; 80% yield; ^1^H NMR (CDCl_3_, 400 MHz) δ: 8.34–8.36(m,2H,Ar-H),8.13(d,1H,*J* = 8 Hz,Ar-H), 7.89(s,1H,Ar-H), 7.75(d,1H,*J* = 8 Hz,Ar-H), 7.60(t,1H,*J* = 8 Hz,Ar-H), 7.36–7.44(m,4H,Ar-H), 7.21(s,1H,Ar-H), 6.95–6.98(m,1H,Ar-H), 3.91(s,6H,N-CH_3,_O-CH_3_). MS: 382.41 m/z = 383.05 [M + 1]^+^.

#### 4.1.8. 2-(4’-chloro-[1,1’-biphenyl]-3-yl)-5-(1-methyl-1H-indazol-3-yl)-1,3,4-oxadiazole (**5c**)

Off-whitesolid; mp 161–165 °C; 82% yield; ^1^H NMR (CDCl_3_, 400 MHz) δ: 8.33–8.35(m,2H,Ar-H), 8.13(d,1H,*J* = 8 Hz,Ar-H), 7.89(s,1H,Ar-H), 7.71(d,1H,*J* = 8 Hz,Ar-H), 7.59–7.62(m,2H,Ar-H), 7.47(d,2H,*J* = 8 Hz,Ar-H), 7.41–7.36(m,3H,Ar-H), 3.92(s,3H,N-CH_3_). MS: 386.83 m/z = 388.00 [M + 1]^+^. 

#### 4.1.9. 2-(1-methyl-1H-indazol-3-yl)-5-(3-(naphthalen-1-yl)phenyl)-1,3,4-oxadiazole (**5d**)

Off-white; mp 163–166 °C; 81% yield; ^1^H NMR (CDCl_3_,400 MHz)δ: 8.33(d,*J* = 8.0 Hz,1H,Ar-H), 8.25–8.27(m,1H,Ar-H), 7.90–7.96(m,3H,Ar-H), 7.85(s,1H,Ar-H),7.67(d, *J* = 8.0 Hz,2H,Ar-H), 7.46–7.60(m,4H,Ar-H), 7.32–7.42(m,3H,Ar-H), 3.89(s,3H,N-CH_3_); MS:402.45 m/z = 403.05 [M + 1]^+^. 

#### 4.1.10. 2-(1-methyl-1H-indazol-3-yl)-5-(2’-methyl-[1,1’-biphenyl]-3-yl)-1,3,4-oxadiazole (**5e**)

White solid;mp 160–164 °C; 79% yield;^1^H NMR (CDCl_3_, 400 MHz) δ: 8.30–8.33(m,1H,Ar-H), 8.14(d,1H,*J* = 8 Hz,Ar-H),7.85(s,1H,Ar-H), 7.58(t,1H,*J* = 8 Hz,Ar-H),7.40(d,1H,*J* = 4 Hz,Ar-H) 7.29–7.42(m,7H,Ar-H), 3.90(s,3H,N-CH_3_), 2.31(s,3H,C-CH_3_), MS:366.42 m/z = 367.06 [M + 1]^+^. 

#### 4.1.11. 2-(1-methyl-1H-indazol-3-yl)-5-(3-(6-methylpyridin-3-yl)phenyl)-1,3,4-oxadiazole (**5f**)

Off-whitesolid; mp 161–166 °C; 83% yield;^1^HNMR(CDCl_3_,400 MHz) δ: 8.80(s,1H,Ar-H), 8.30(d,2H,*J* = 8 Hz,Ar-H), 8.11(d,1H,*J* = 8 Hz,Ar-H), 7.84–7.87(m,1H,Ar-H), 7.84(s,1H,Ar-H), 7.69(d,1H,*J* = 8 Hz,Ar-H), 7.60(t,1H,*J* = 8 Hz,Ar-H), 7.32–7.38(m,3H,Ar-H), 3.87(s,3H,N-CH_3_), 2.62(s,3H,C-CH_3_). ^13^C-NMR: 162.37, 162.30, 157.81, 147.26, 134.99, 130.69, 129.73, 129.58, 125.91, 124.98, 123.41, 123.30, 121.86, 121.31, 109.90, 99.91, 33.46, 24.02. MS:367.40 m/z = 368.05 [M + 1]^+^. 

#### 4.1.12. 2-(1-methyl-1H-indazol-3-yl)-5-(3-(pyrimidin-5-yl)phenyl)-1,3,4-oxadiazole(**5g**)

Dark-brown; mp 230–235 °C; 81% yield; ^1^H NMR (CDCl_3_, 400 MHz) δ: 9.27(s,1H,Ar-H), 9.04(s,1H,Ar-H), 8.29–8.33(m,2H,Ar-H), 8.21(d,1H,*J* = 8 Hz,Ar-H), 7.86(s,1H,Ar-H), 7.66–7.73(m,2H,Ar-H), 7.37(d,3H,*J* = 8 Hz,Ar-H), 3.89(s,3H,N-CH_3_).^13^C-NMR: 162.46, 161.98, 157.94, 154.99, 137.15 (2C), 135.21, 133.40, 130.78, 130.20, 129.53, 127.01, 125.58, 125.07, 124.96, 123.37, 121.94, 121.28, 109.96, 99.78, 33.52. MS: 354.36 m/z = 355.03 [M + 1]^+^. 

#### 4.1.13. 2-(3-(5,6-dimethylpyridin-3-yl)phenyl)-5-(1-methyl-1H-indazol-3-yl)-1,3,4-oxadiazole (**5h**)

Light-brown; mp 161–166 °C; 86% yield; ^1^H NMR (CDCl_3_, 400 MHz) δ: 8.66(s,1H,Ar-H), 8.30(s,1H,Ar-H), 8.11(d,1H,*J* = 8 Hz,Ar-H), 7.87(s,1H,Ar-H); 7.70–7.76(m,2H,Ar-H), 7.59–7.62(m,1H,Ar-H), 7.36–7.38(m,3H,Ar-H), 3.89(s,3H,N-CH_3_), 2.59(s,3H,C-CH_3_), 2.39(s,3H,C-CH_3_). ^13^C-NMR: 162.31 (2C), 156.15, 143.78, 138.41, 137.17, 136.45, 133.53, 130.73, 129.74, 129.55, 125.99, 125.12, 124.97, 123.31, 121.86, 121.32 (2C), 109.91, 99.92, 33.46, 21.75, 19.15.MS:381.43 m/z = 382.08 [M + 1]^+^.

#### 4.1.14. 2-(3-(6-chloro-5-methylpyridin-3-yl)phenyl)-5-(1-methyl-1H-indazol-3-yl)-1,3,4-oxadiazole (**5i**, CHK9)

Off-whitesolid; mp 163–167 °C; 85% yield; ^1^H NMR (CDCl_3_, 400 MHz) δ: 8.57(s,1H,Ar-H), 8.29(s,1H,Ar-H), 8.13(d,1H,*J* = 8 Hz,Ar-H), 7.83(d,2H,*J* = 12 Hz,Ar-H), 7.59–7.69(m,2H,Ar-H), 7.36(d,3H,*J* = 4 Hz,Ar-H), 3.88(s,3H,N-CH_3_), 2.47(s,3H,C-CH_3_). ^13^C-NMR: 162.36, 162.20, 151.10, 145.14, 137.84 (2C), 130.71, 129.84 (2C), 129.59, 126.30 (2C), 125.22, 125.04 (2C), 123.33, 121.88, 121.29, 109.93, 99.84, 33.47, 19.71. MS:401.85 m/z = 403.01 [M + 1]^+^.

#### 4.1.15. 2-(3-(6-fluoro-5-methylpyridin-3-yl)phenyl)-5-(1-methyl-1H-indazol-3-yl)-1,3,4-oxadiazole (**5j**)

Brown; mp 234–239 °C; 86% yield;^1^H NMR (CDCl_3_, 400 MHz) δ: 8.30–8.32(m,1H,Ar-H), 8.22(d,1H,*J* = 8 Hz,Ar-H), 8.12(d,1H,*J* = 8 Hz,Ar-H), 8.10(s,1H,Ar-H), 7.88(s,1H,Ar-H), 7.65(t,1H,*J* = 8 Hz,Ar-H), 7.48(d,1H,*J* = 8 Hz,Ar-H), 7.35–7.43(m,3H,Ar-H), 3.91(s,3H,N-CH_3_), 2.26(s,3H,C-CH_3_); ^13^C-NMR: 163.95, 162.44, 153.06, 144.31, 144.15, 138.95, 137.04, 131.23, 130.77, 129.36, 126.36, 126.59, 126.56, 123.38, 122.42, 122.38, 121.93, 121.28, 109.98, 99.81, 33.52, 14.12. 

#### 4.1.16. 3’-(5-(1-methyl-1H-indazol-3-yl)-1,3,4-oxadiazol-2-yl)-[1,1’-biphenyl]-3-carbaldehyde (**5k**)

Brown; mp 159–163 °C; 87% yield; ^1^H NMR (CDCl_3_, 400 MHz) δ: 10.11(s,1H,-CHO), 8.37(s,1H,Ar-H), 8.32(t,1H,*J* = 8 Hz,Ar-H), 8.15(m,2H,Ar-H), 7.92(t,2H,*J* = 12 Hz,Ar-H), 7.87(s,1H,Ar-H), 7.77(d,1H,*J* = 8 Hz,Ar-H), 7.61–7.68(m,3H,Ar-H), 7.34–7.38(m,3H,Ar-H), 3.89(s,3H,N-CH_3_). ^13^C-NMR: 192.17, 162.41, 162.33, 140.95, 140.57, 137.16, 136.98, 133.08, 130.74, 129.83, 129.70, 129.26, 128.10,126.10, 125.18, 125.12, 125.04, 123.32, 121.87, 121.34, 109.92, 99.87, 33.80.

#### 4.1.17. N-cyclopentyl-3’-(5-(1-methyl-1H-indazol-3-yl)-1,3,4-oxadiazol-2-yl)-[1,1’-biphenyl]-3-carboxamide (**5l**)

Brown; mp 184–190 °C; 85% yield;^1^H NMR (CDCl_3_, 400 MHz) δ: 8.32(s,1H,Ar-H), 8.06–8.10(m,2H,Ar-H), 7.85(s,1H,Ar-H), 7.71–7.77(m,3H,Ar-H),7.50–7.59(m,2H,Ar-H), 7.36–7.37(m,1H,Ar-H), 7.14(d,1H,*J* = 8 Hz,Ar-H), 7.02(d,1H,*J* = 8 Hz,Ar-H), 4.32–4.47(m,2H,N-CH), 3.87(s,3H,NH-C=O), 1.95–2.23(m,8H,-CH_2_ and -CH). ^13^C-NMR: 167.64, 167.35, 162.70, 157.40, 141.38, 140.60, 137.33, 135.78, 130.97, 130.17, 129.73, 129.28, 126.00, 125.42, 123.48, 122.04, 121.49, 118.99, 117.75, 114.88, 110.07, 99.99, 52.09, 33.59, 33.34, 33.29, 24.04, 23.95.

#### 4.1.18. 2-(4’-fluoro-[1,1’-biphenyl]-3-yl)-5-(1-methyl-1H-indazol-3-yl)-1,3,4-oxadiazole (**5m**)

Off-whitesolid; mp 160–165 °C; 85% yield; ^1^H NMR (CDCl_3_, 400 MHz) δ: 8.30–8.33(m,1H,Ar-H), 8.06(d,1H,*J* = 4 Hz,Ar-H), 7.81(s,1H,Ar-H), 7.54–7.67(m,4H,Ar-H), 7.32–7.36(m,3H,Ar-H), 7.16(t,2H,*J* = 8 Hz,Ar-H), 3.86(s,3H,N-CH_3_). ^13^C-NMR: 164.15, 162.69, 162.37, 141.21, 137.30, 130.73, 129.81, 129.63, 129.00, 128.92, 125.52, 125.29, 125.21, 125.06, 123.43, 121.96, 121.51, 116.07, 115.86, 110.03, 100.17, 33.57. 

### 4.2. Biology

The human lung cancer (A549), breast cancer (MCF-7), skin cancer (A375), liver (HepG2, Huh-7), kidney cancer (ACHN, A498) cell lines and mouse cancer skin (B16-F10) and Lewis lung carcinoma (LLC) cells were procured from the National Centre for Cell Science, Pune, India. The 3-(4,5-dimethylthiazole-2yl)-2,5-diphenyltetrazolium bromide (MTT), propidium iodide, protease inhibitor cocktail, and anti-mouse/rabbit IgG antibodies were obtained from Sigma Aldrich (St. Louis, MO, USA). Dulbecco’s modified Eagle media (DMEM), antibiotic-antimycotic solution, trypsin-EDTA solution, fetal bovine serum (FBS) were procured from Invitrogen (Gibco), Waltham, MA, USA. p-STAT3, STAT3, p-JAK2, JAK2 antibodies were purchased from Cell Signalling Technologies, Danvers, MA, USA. p-STAT5 and STAT5 antibodies were procured from Santa Cruz Biotechnology (Santa Cruz, CA, USA). Caspase-3, cleaved PARP, Bax, Bad, Bcl-2, Cytochrome C antibodies, and Annexin V–FITC staining kit were purchased from BD Bioscience, (Franklin Lakes, NJ, USA). TUNEL vision kit and DAPI were obtained from Thermofisher Scientific, Waltham, MA, USA. All other chemicals used in the current study were of molecular and analytical grade. Cell culture plastic wares were from corning (New York, NY, USA), Sigma (St. Louis, MO, USA) and Eppendorf (Hamburg, Germany). All bright field and fluorescence images were taken in EVOS FL cell imaging, Thermo Scientific, (Waltham, MA, USA), and results were assessed and analyzed by ImageJ software. All the gel and blotting images were documented using Bio-rad Gel Documentation TM XR + Imaging system and quantified using densitometric analysis.

#### 4.2.1. Animal Cell Culture and Growth Inhibition Studies

A549, MCF-7, A375, HepG2, HuH-7, ACHN, A498, B16-F10, and LLC cells were cultured in DMEM, enriched with 10% FBS with 1% antibiotic-antimycotic solution and maintained in a humidified atmosphere containing 5% CO_2_ at 37 °C. The stock solution of new compounds was prepared in DMSO and diluted the stock solution with a cell culture medium to the desired concentration. In brief, cancer cells were seeded at 4 × 10^3^ cells per well into 96-well plates, cultured overnight, and treated with increasing concentration of newly synthesized compounds (0, 2, 5, 10, 25, and 50 µM). The ability of newly synthesized molecules to inhibit cancer cell proliferation was assessed using trypan blue dye exclusion, MTT assay, and lactate dehydrogenase (LDH) release assay, as reported previously [58]. Compound CHK9 consistently showed good cytotoxicity towards lung cancer cells, and therefore, the underlying molecular mechanism of CHK9 was evaluated towards human lung cancer cells. 

#### 4.2.2. Flow Cytometric Analysis

The A549 (4 × 10^3^) cells were seeded in a cell-culture vessel and treated with varying concentrations of CHK9 (0, 3, and 5 μM) for 48 h along with appropriate vehicle control as described earlier [59,60]. In brief, CHK9-treated cells were incubated with propidium iodide and RNase for 30 min and subjected to FACS analysis and apoptosis was analyzed using WinMDI version 2.9 software [61].

#### 4.2.3. Annexin V Staining 

The A549 (4 × 10^3^) cells were seeded in a cell-culture vessel and treated with varying concentrations of CHK9 (0, 3, and 5 μM) for 48 h along with appropriate vehicle control as described earlier. CHK9-treated cells stained with Annexin V staining-FITC Fluorescence Microscopy Kit as per the manufacturer’s instructions and images were documented.

#### 4.2.4. TUNEL Assay 

The A549 cells were cultured on poly-L-lysine pre-coated coverslips and exposed with CHK9 (0, 3 and 5 µM) for 48 h. Cells were fixed with 4% paraformaldehyde for 15 min and permeabilized by 0.25% Triton X-100 for 20 min, and nick end labeling (TUNEL) was performed as per the manufacturer’s instructions [62].

#### 4.2.5. DAPI Staining 

Cells were cultured on poly-l-lysine pre-coated coverslip and treated with or without CHK9 treatment. Further cells were fixed, permeabilized, and stained with DAPI for 10 min, and photographs were documented.

#### 4.2.6. Preparation of Cell Lysate

The whole-cell lysate was prepared as described earlier [63,64]. In brief, cells treated with CHK9 or DMSO (0, 3 and 5 μM) were harvested and whole-cell extract was prepared using radioimmunoprecipitation assay buffer (RIPA buffer) (100 mM Tris pH-7.5,1% Triton X-100,0.1% SDS, 140 mM NaCl, 0.5% sodium deoxycholate, 5 mM EDTA, 0.5 mM PMSF and protease inhibitor cocktail. The total protein content was quantified by the Bradford method using the Nanodrop- biospectrophotometer (Eppendorf, Germany).

#### 4.2.7. STAT3 Luciferase Reporter Assay

The effect of CHK9 on constitutive activation of STAT3-driven luciferase gene expression in lung cancer cells was determined as reported earlier [65,66]. STAT3 targeted elements conjugated to a luciferase gene were transfected with wild-type or dominant-negative STAT3. The transfected cells were incubated with indicated doses of CHK9 up to 24 h. Luciferase activity was measured with a Tecan (Durham, NC, USA) plate reader and normalized to β-galactosidase activity. All luciferase experiments were done in triplicate and repeated twice.

#### 4.2.8. Immunoblot Analysis 

The cell lysates were separated on a precast SDS polyacrylamide gel, transferred to PVDF membrane, probed with appropriate antibodies and detected. The bands were densitometrically analyzed and documented as described earlier [67,68].

#### 4.2.9. In silico Studies

Discovery Studio 2.5 version of Insight II from the Accelrys program was used for molecular docking analysis as described earlier [69]. The structure-based analyses were subjected to STAT3β homodimer (PDB ID: 1BG1) [70]. CDOCKER protocol of Discovery Studio was used for this study, and the STAT3 monomer was cleaned, and the SH2 domain of STAT3 active site was identified. The CHARMM force field was used for energy minimization, followed by molecular docking analyses. Each energy-minimized final docking position was studied using the interaction scoring function in the Ligand Fit module of Discovery Studio. 

#### 4.2.10. In Vivo Experiments

The BALB/c mice (27–30 g) were used throughout the study and maintained as per CPCSEA guidelines with ethical clearance. In vivo efficacy of CHK9 was tested using LLC induced lung cancer model in BALB/c mice [71]. In brief, LLC cells were cultured and injected intravenously (1 × 10^6^) in 100 μL of PBS into the lateral tail veins of 8-week-old female mice. Each experiment was comprised of three groups of animals comprising six mice per group. Group I animals served as tumor control. Group II and III animals received an intraperitoneal injection of CHK9 (10 and 20 mg/kg body weight, respectively, as determined by LD_50_ studies) on alternate days. On the 30th day after tumor implantation, animals were sacrificed, and lung tissues were collected for further processing and histological examination. Tumor nodules were quantified by inspection of the pleural surface of all lobes of the tumor-bearing lung stained with hematoxylin and eosin (H&E) and photographed. Blood samples of experimental animals were collected by the cardiac puncture. The levels of markers enzymes such as serum glutamate oxaloacetate transaminase (SGOT) and serum glutamate pyruvate transaminase (SGPT), and other biochemical parameters such as creatinine, platelet count, red blood cells (RBCs), and white blood cells (WBCs) were quantified.

## Figures and Tables

**Table 1:** List of newly synthesized oxadiazoles.

Entry	Oxadiazole	Boronic acid	Biphenyl-1,3,4-oxadiazole
1		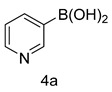	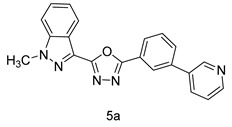
2		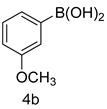	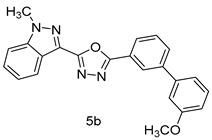
3	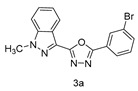	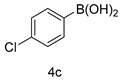	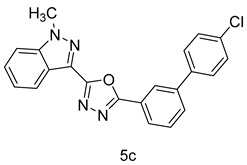
4		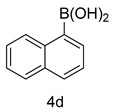	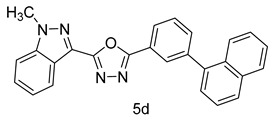
5		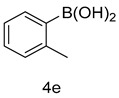	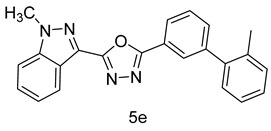
6		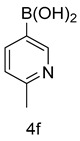	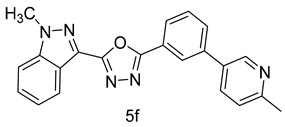
7		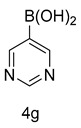	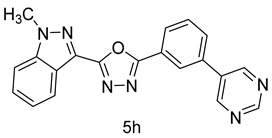
8		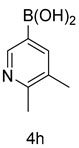	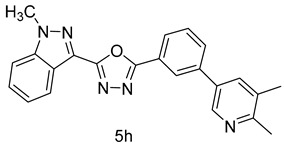
9		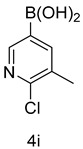	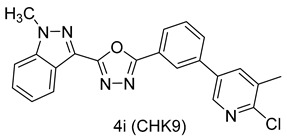
10		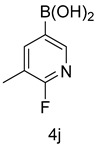	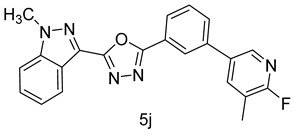
11		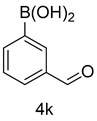	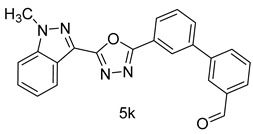
12		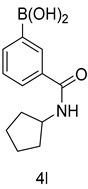	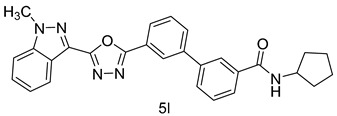
13		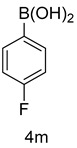	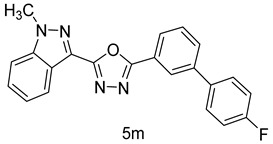

## Supplementary Materials

Supplementary materials can be found at https://www.mdpi.com/2227-9059/8/9/368/s1.

## Statistical Analysis

Values were represented as the mean ± standard deviation (SD). Statistical significance was evaluated by one-way analysis of variance (ANOVA) followed by a 2-tailed 13 Student’s *t*-test. MS Excel 8.1 version software was used for data analysis, and statistically significant values were expressed as * *p* < 0.05 and ** *p* < 0.01.
