# Supplementary material for: Novel 1,3,4-oxadiazole Targets STAT3 Signaling to Induce Antitumor Effect in Lung Cancer
Source: Biomedicines. 2020 Sep 21;8(9):368. doi: 10.3390/biomedicines8090368 (PMC7555749; doi:10.3390/biomedicines8090368)
Supplement: Supplementary file 1 [file biomedicines-08-00368-s001.pdf]

Novel 1,3,4-oxadiazole Targets STAT3 Signaling to Induce Antitumor Effect in Lung Cancer

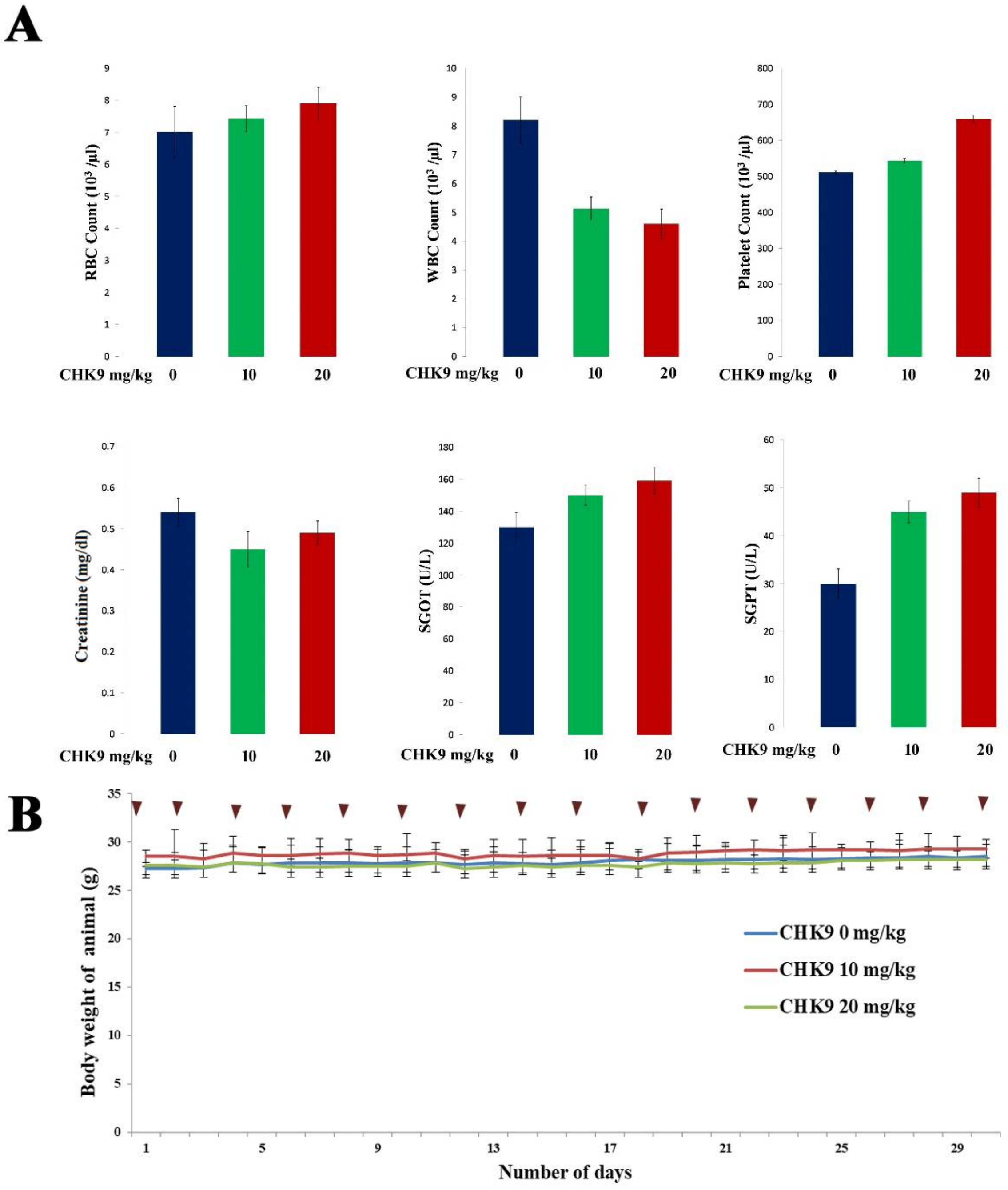

Supplementary Figure 1. CHK9 inhibits colonization of LLC lung cancer cells in animal models without significant side effects. LLC cells were injected through the tail vein into the BALB/c mice. The mice received 10 and 20 mg/kg CHK9 intraperitoneally every other day for five weeks as indicated by arrowheads. (A) The hematology and biochemistry parameters of blood from treated and untreated mice. In the hematology parameters, white blood cells (WBCs), red blood cells (RBCs), and platelets were tested. In biochemistry parameters, glutamate oxaloacetate transaminase (GOT) and glutamate pyruvate transaminase (GPT) were used as indicators of liver function, and creatinine

as indicators of renal function. (B) Bodyweight of LLC injected mice of different experimental groups were shown.
